# Feasibility and Effectiveness of Provider Initiated HIV Testing and Counseling of TB Suspects in Vizianagaram District, South India

**DOI:** 10.1371/journal.pone.0041378

**Published:** 2012-07-23

**Authors:** Shanta Achanta, Ajay M. V. Kumar, Sharath Burugina Nagaraja, Jyoti Jaju, Srinivas Rao Motta Shamrao, Ramakrishna Uppaluri, Rama Rao Tekumalla, Devesh Gupta, Ashok Kumar, Srinath Satyanarayana, Puneet K. Dewan

**Affiliations:** 1 Office of the World Health Organization (WHO) Representative to India, New Delhi, India; 2 Central TB Division, Directorate General of Health Services, Ministry of Health and Family Welfare, Government of India, New Delhi, India; 3 State TB Cell, Directorate of Health Services, Hyderabad, Andhra Pradesh, India; 4 Andhra Pradesh State AIDS Prevention and Control Society, Directorate of Health Services, Hyderabad,Andhra Pradesh, India; 5 District TB Centre, Directorate of Health Services, Vizianagaram, Andhra Pradesh, India; 6 International Union against Tuberculosis and Lung Diseases (The Union), South East Asia Regional Office, New Delhi, India; McGill University, Canada

## Abstract

**Background:**

Though internationally recommended, provider initiated HIV testing and counseling (PITC) of persons suspected of tuberculosis (TB) is not a policy in India; HIV seroprevalence among TB suspects has never been reported. The current policy of PITC for diagnosed TB cases may limit opportunities of early HIV diagnosis and treatment. We determined HIV seroprevalence among persons suspected of TB and assessed feasibility and effectiveness of PITC implementation at this earlier stage in the TB diagnostic pathway.

**Methods:**

All adults examined for diagnostic sputum microscopy (TB suspects) in Vizianagaram district (population 2.5 million), in November-December 2010, were offered voluntary HIV counseling and testing (VCT) and assessed for TB diagnosis.

**Results:**

Of 2918 eligible TB suspects, 2465(85%) consented to VCT. Among these, 246(10%) were HIV-positive. Of the 246, 84(34%) were newly diagnosed as HIV (HIV status not known previously). To detect a new case of HIV infection, the number needed to screen (NNS) was 26 among ‘TB suspects’, comparable to that among ‘TB patients’. Among suspects aged 25–54 years, not diagnosed as TB, the NNS was 17.

**Conclusion:**

The seroprevalence of HIV among ‘TB suspects’ was as high as that among ‘TB patients’. Implementation of PITC among TB suspects was feasible and effective, detecting a large number of new HIV cases with minimal additional workload on staff of HIV testing centre. HIV testing of TB suspects aged 25–54 years demonstrated higher yield for a given effort, and should be considered by policy makers at least in settings with high HIV prevalence.

## Introduction

According to World Health Organization’s (WHO) global TB report 2010, Tuberculosis (TB) accounted for 25% of all deaths among people living with HIV/AIDS (PLHIV) in 2009 [1]. Unless HIV-infected TB patients are diagnosed early and linked to both TB and HIV treatment, mortality will remain high. To facilitate early and enhanced diagnosis of HIV-infected TB patients, WHO recommends intensified TB case finding at all HIV care settings and ‘provider initiated HIV testing and counseling’ (PITC) for patients with diagnosed and presumptive TB [2].

The recommendation of PITC for all TB suspects is based on study findings from sub-Saharan African countries, which have shown a very high HIV prevalence among persons being evaluated for TB (TB suspects), sometimes even higher than HIV prevalence among TB patients [3], [4]. These findings from HIV endemic settings cannot, however, be generalized to lower HIV burden settings such as India. With paucity of previously published literature on this issue from India, it remains unclear if TB suspects indeed have a similar HIV prevalence as compared to diagnosed TB patients, or even if HIV prevalence among TB suspects is higher than that found in the general community. Hence, despite recommendations from WHO and International Standards of TB Care (ISTC) [5], in India routine HIV testing is offered only to ‘TB patients’ and not to all ‘TB suspects’.

The strategy of routine HIV testing of all TB suspects offers the potential for early HIV diagnosis and treatment with consequent reduction of morbidity and mortality. Evidence showing that HIV prevalence among TB suspects is relatively high and PITC implementation among them is feasible might guide policy makers seeking to improve TB and HIV care [6].

We conducted this study to assess the feasibility and effectiveness of routinely offering HIV counseling and testing to all TB suspects in a relatively high-HIV prevalence district of South India. The four specific objectives were: (1) to assess the proportion of ‘TB suspects’ and ‘TB patients’ tested for HIV and found HIV positive, (2) to assess the number of newly-detected cases of HIV infection detected as a result of this strategy, (3) to assess the number needed to screen (NNS) to find one new case of HIV among ‘TB patients’ and ‘TB suspects’, and (4) to assess the additional workload at HIV testing centers due to this strategy.

## Methods

### Study Design

This was a cross sectional study conducted among TB suspects attending the designated microscopic centers (DMC) in the district for diagnosis of TB.

### Setting

The study was conducted in Vizianagaram, a coastal district in the state of Andhra Pradesh, India with a population of 2.5 million. This district has been prioritized for HIV services by National AIDS Control Programme (NACP) as the HIV prevalence among antenatal clinic attendees has been consistently exceeding 1% over the past 5 years [7]. HIV diagnostic and treatment services are offered free of cost through a network of 70 HIV testing centers and one Anti Retro-viral Therapy(ART) centre as per national guidelines [8].

Under the ambit of Revised National TB Control Programme (RNTCP), TB diagnosis and treatment services are offered through the primary health care system of the district. TB suspects, defined as anybody with a cough of two weeks or more with or without other symptoms (For PLHIV, cough of any duration is considered as TB suspect) are examined at one of the 31 DMC for sputum smear microscopy. All the microscopy centers in the district are covered under external quality assurance which is a crucial component of the quality assurance mechanism adopted by RNTCP. Those who are diagnosed as TB patients are treated with fully intermittent short course chemotherapy administered under direct observation (DOT) and registered in one of the six programme management units as per national guidelines [9].

As per the national framework of joint TB/HIV collaborative activities [10], HIV testing is offered routinely to all the TB patients treated under RNTCP and those who are found HIV-infected are provided cotrimoxazole prophylaxis and referred to ART centre for assessment of ART eligibility and initiation. During 2010, 18,799 TB suspects underwent sputum smear microscopy and 3,760 TB patients were registered for treatment [11]. Of the TB patients registered, 3,619 (96%) were ascertained for HIV status and 320 (9%) were found to be HIV-infected [11].

### Study Population and Study Period

The study was conducted during the period from October 2010 to January 2011. All the adult TB suspects (more than 18 years of age) examined for diagnostic smear microscopy at the DMCs of Vizianagaram district from November 1 to December 31, 2010 formed the study population.

### Sample Size and Sampling

Assuming 7% HIV prevalence among TB suspects, an absolute precision of 1% with 95% confidence, a possible attrition rate of 10%, the sample size was calculated to be 2744. Considering the number of TB suspects examined per month in the district, it was decided to enroll all the eligible TB suspects for a period of two months.

### Data Collection and Data Validation

All the adult TB suspects attending the DMC were provided information on the study by the laboratory technician (LT) trained for the purpose. Those who consented to participate in the study were referred to the co-located HIV testing centre for HIV testing and counseling. As per national guidelines, TB suspects with HIV status already known were not tested again. Those with a prior known positive HIV status or with a HIV negative result within the previous six months were referred to as those with ‘prior HIV status known’. The study participants were tracked for a month by the TB treatment supervisors of the programme, to assess if they were diagnosed as smear negative pulmonary TB or extra pulmonary TB. The information on the following variables – name of DMC, age, sex, sputum smear result, whether diagnosed as TB, type of TB, HIV status, - were extracted into a pre-tested structured data collection format by LT in co-ordination with the counselor at HIV testing centre. All the data collection formats were checked for completeness and consistency by the TB laboratory supervisors of the programme once a week and by the principal investigator once in a fortnight. All the staff involved in data collection, data validation and data entry were trained in carrying out the respective procedures, using the study protocol and data collection formats.

### Definitions of Key Outcomes

We calculated the following key indicators – i) Proportions of ‘TB suspects’ and ‘TB patients’ with known HIV status, ii) Proportions of ‘TB suspects’ and ‘TB patients’ found HIV positive, iii) The number (proportion) of all HIV cases diagnosed newly as the result of the strategy of ‘PITC of TB suspects’, iv) Number needed to screen (NNS) to diagnose an additional case of HIV, separately among ‘TB patients’ and ‘TB suspects not diagnosed as TB’, v) The average increase in daily workload at the HIV testing centers calculated centre wise by dividing the total number of TB suspects who underwent HIV testing by the average number of working days.

### Data Entry, Analysis and Reporting

The data were entered twice, independently by two data entry operators into a pre-designed data entry form with inbuilt checks to minimize data entry errors using Epi Data entry software [12]. Both the databases were compared and discrepancies were resolved by referring to the original data collection formats. All analyses was done using Epi Data analysis software [13]. NNS is the reciprocal of the proportion of newly-detected HIV infection, i.e. excluding those persons with previously-known HIV status. Those with prior known HIV status were excluded and the number of TB suspects needed to be screened to diagnose one new case of HIV infection was calculated to determine the yield by the PITC strategy. Chi-square tests were used for comparing proportions and ‘p’ value of less than 0.05 was considered as statistically significant. We have adhered to STROBE guidelines for reporting of observational studies in writing this manuscript.

### Ethics Considerations

The study was approved by the Ethics Advisory Group of the International Union of TB and Lung disease (The Union) and National Tuberculosis Institute, Bangalore. Administrative clearances were obtained from state and central authorities for conducting this study. A written informed consent was taken from each patient and confidentiality was assured as data collection formats were maintained securely by programme staff and electronic databases contained no personal identifiers.

## Results

Of the 3,232 TB suspects examined for sputum smear microscopy, 314 were aged less than 18 years and excluded from the study. Of the remaining 2,918 adult TB suspects, 2,465 (85%) consented for HIV testing.

The demographic and clinical characteristics of study participants who consented for HIV testing as compared to those who did not are shown in Table 1. Those who did not consent for HIV testing were more likely to belong to older age groups and less likely to have HIV.

**Table 1:** Demographic and clinical characteristics of the study population, Vizianagaram district, India, November-December 2010.

Characteristic	TB suspects who did not consentfor HIV testing N (%)	TB suspects who consented forHIV testing N (%)	P value(df)
Total	453 (100)	2465 (100)	
Age (years)			
18–24	43 (10)	220 (9)	<0.01 (5)
25–34	71 (16)	417 (17)	
35–44	86 (19)	569 (23)	
45–54	79 (17)	561 (23)	
55–64	87 (19)	473 (19)	
≥65	87 (19)	225 (9)	
Sex			
Male	317 (70)	1648 (67)	0.19 (1)
Female	136 (30)	817 (33)	
TB status			
TB patients	27 (6)	381 (16)	<0.01 (1)
TB suspects without TB	426 (94)	2084 (84)	

TB – Tuberculosis; HIV – Human immunodeficiency virus; df-degrees of freedom.

The proportions of TB suspects who were tested for HIV and found to be HIV infected are shown in Table 2. Of the 2465 (85%) of TB suspects tested for HIV, 246 (10%) were HIV infected. The HIV prevalence was found to be higher among the age-group 25–44, females and ‘TB suspects without TB’.

**Table 2:** HIV prevalence among TB suspects examined for diagnostic smear microscopy, Vizianagaram district, India, November-December 2010.

Characteristic	Number of TB suspects examinedfor smear microscopy	Number (%) of TB suspects with known HIV status	Number (%) HIV Positive
Total	2918	2465 (84.5)	246 (10.0)
Age (years)			
18–24	263	220 (83.7)	15 (6.8)
25–34	488	417 (85.5)	86 (20.6)
35–44	655	569 (86.9)	105 (18.5)
45–54	640	561 (87.7)	31 (5.5)
55–64	560	473 (84.5)	8 (1.7)
≥65	312	225 (72.1)	1 (0.4)
Sex			
Male	1965	1648 (83.9)	147 (8.9)
Female	953	817 (85.7)	99 (12.1)
TB status			
TB patients	408	381 (93.4)	31 (8.1)
TB suspects without TB	2510	2084 (83.0)	215 (10.3)

TB–Tuberculosis; HIV–Human immunodeficiency virus.

The newly detected number of HIV patients is shown in Table 3. Among the 246 HIV-infected patients identified in the study population, 162 (67%) had their HIV status known prior to the study and 84 (34%) were diagnosed newly as part of the study. Of the 84 newly-detected cases of HIV among the population of TB suspects, 70 (83%) were among those not ultimately diagnosed with TB, and only 14 (17%) were subsequently diagnosed with TB. These are the additional number of HIV cases detected by the strategy of offering HIV testing to all TB suspects. Excluding those with prior known HIV status, the number needed to screen (NNS) to find an additional HIV case was found to be 25 among ‘TB patients’ and was comparable to that among ‘TB suspects without TB’ (Table 4).

**Table 3:** Newly detected HIV infections among TB patients and TB suspects, Vizianagaram district, India, November-December 2010.

Category	Prior HIV status Known		Newly detected HIV		Proportionate increase in the number of new cases of HIV
	HIV Positive [a]	HIV Negative [b]	HIV Positive [c]	HIV Negative [d]	[c]/[a+c]
TB patients (n = 381)	17	16	14	334	45%
TB suspects without TB(n = 2084)	145	85	70	1784	33%
Total	162	101	84	2118	34%

TB – Tuberculosis; HIV – Human immunodeficiency virus.

**Table 4:** Number needed to screen to find an additional case of HIV, by strategy, Vizianagaram district, India, November-December 2010.

Strategy	Number with Prior HIV status Unknown	Number (%) HIV Positive	Number Needed to Screen (NNS)
HIV testing for all TB patients	348	14 (4.0)	25
HIV testing for TB suspects excluding TB patients	1854	70 (3.7)	26
HIV testing for all TB suspects including TB patients	2202	84 (3.8)	26
HIV testing of all TB suspects in the age group 25–54 years	1331	75 (5.6)	17

TB –Tuberculosis; HIV –Human immunodeficiency virus; NNS-Number needed to screen.

The average increase in daily workload at the HIV testing centers due to the implementation of this strategy is shown in Table 5. Most of the centers (27/31) had minimal increase in workload with only 1–2 extra clients to be counseled and tested in a day.

**Table 5:** Increase in workload at HIV testing centers due to strategy of ‘routine HIV testing of TB suspects’, Vizianagaram district, India, November-December 2010.

Average increase in number of clientstested for HIV per day	Number of ICTCs (N = 31)
<3	27
3–5	3
6–10	1

HIV-Human immunodeficiency virus; ICTC-Integrated counseling and testing centre.

## Discussion

PITC for TB suspects effectively detected a large number of additional HIV cases and could be feasibly implemented with the existing resources within the programme. About 33% of the newly-diagnosed instances of HIV infection in the overall population of TB suspects would likely have been missed if HIV testing was applied after TB diagnosis, i.e. to TB cases only. We found that overall 10% of the TB suspects tested for HIV were HIV-infected, indicating that HIV prevalence among TB suspects is higher than that reported among general population in the state of Andhra Pradesh (0.96%) [14], comparable to HIV prevalence among TB patients (9%) [11].

This is one of the first studies from India examining the effectiveness and feasibility of implementing the strategy of PITC of TB suspects under programmatic settings. Another study conducted in Mandya district of state of Karnataka in South India showed a HIV prevalence of 7% among TB suspects with nearly 40% of the HIV cases newly diagnosed as a result of the strategy of routine offer of HIV testing [15]. Similar studies from other parts of the world, mainly from African countries, indicate that HIV prevalence among TB suspects varied from 27% to 63% and was as high as or even higher than among TB patients [3], [4], [16], [17], [18], [19]. However, the very high levels of HIV among TB suspects in African countries are reflective of the high levels of HIV in their general population.

Among the 246 HIV-infected patients identified in the study population, 84 (34%) were newly diagnosed during the study, but most of these (70) were among TB suspects not subsequently diagnosed as TB. These 70 newly-detected instances of HIV infection reflect the additional increase in detection of HIV-infected cases because of offering HIV testing to all TB suspects. The NNS of TB suspects to find one additional instance of HIV infection indicated that screening TB suspects is as productive as screening TB patients in terms of diagnosing new HIV infected persons. Clearly, PITC among TB suspects is a very effective strategy of HIV case finding in an easily accessible population.

This study also demonstrated the feasibility of implementation of PITC among TB suspects. We found that a great majority of the TB suspects (about 85%) consented for HIV testing indicating very high acceptance levels for HIV testing among the TB suspects. Further, centre-wise workload analysis indicated that most of the HIV testing centers (27/31) had a minimal increase in additional workload with only 1–2 extra clients to be counseled and tested in a day. Hence this strategy can be implemented with the existing human resources and almost negligible additional burden on the health staff delivering services. If the resource investment were still felt to be too great, limiting HIV testing of TB suspects to adults could also be considered. A sub-analysis among TB suspects aged 25–54 years indicated that examining as few as 17 TB suspects in this age group would yield one additional case of HIV. Since, about 90% of all HIV-infected individuals were in this age group and could be detected by testing only 60% of TB suspects; selectively adopting the strategy of PITC among TB suspects aged 25–54 years would be highly resource-optimizing.

There are two key considerations and reasons for policy makers to exercise caution before implementing this strategy more widely. Firstly, the high HIV testing rates among TB suspects in our study was hugely facilitated by the widespread availability of HIV testing services co-located with the DMCs. The easy availability of HIV-testing services has been shown to be closely associated with HIV testing uptake [20]. Co-location of HIV testing services at all DMCs is seemingly a pre-requisite for successful implementation of this strategy. Secondly, the total number of HIV tests performed can increase substantially as a result of this strategy and national programme needs to plan for enhanced procurement and supply chain management of HIV testing kits before launching this strategy.

There are other positive implications of this strategy to consider. Firstly, the early diagnosis of HIV-infected TB patients and linkage to HIV care and support can potentially be life saving. Secondly, this strategy identifies HIV-infected individuals in whom TB has been ruled out and who are thus eligible for Isoniazid preventive therapy (IPT) as per WHO guidelines; linking these individuals to early antiretroviral therapy (ART) and IPT can have a substantial impact in preventing TB in this vulnerable group.

There were some limitations to this study. Firstly, about 15% of the TB suspects did not consent for HIV testing. Non-consenting TB suspects were more likely to belong to older age group who are in general less likely to be HIV infected; hence this could have overestimated the overall HIV prevalence. However, if we assume all the untested patients were HIV negative, then the minimum HIV prevalence among TB suspects would still be 8.5%. Thus our enrollment rate did not change the interpretation of study findings. Secondly, the study captured only pulmonary TB suspects attending state-run designated microscopy centres. We were not able to assess HIV prevalence among isolate extra-pulmonary TB suspects who were not eligible for sputum examination, or TB suspects evaluated at private laboratories outside national programme. Furthermore, it was beyond the scope of this study to evaluate if offering an early opportunity for HIV testing among TB suspects actually translated into early initiation of HIV care and support and the expected morbidity and mortality benefits; longer term HIV care outcomes should be the subject of future operational research.

### Translating Research Findings into Policy

Acknowledging the strong evidence found as part of this study and another study conducted simultaneously in another district of South India [15], the National Technical Working Group (NTWG) of TB/HIV collaborative activities took a policy decision to implement PITC among TB suspects in high HIV settings (states of Karnataka, Andhra Pradesh, Tamil Nadu, Maharashtra, Manipur and Nagaland) in India. It was further recommended that PITC among TB suspects be piloted in 1–2 high prevalence states (and select districts in other high prevalent states, at all microscopy centers with co-located HIV testing facility) for a period of 3–6 months with mechanisms for recording and reporting to finalize the operational guidance before scale-up to other high HIV settings.

Considering a high yield of HIV among TB suspects in high prevalent settings, which was hitherto unexpected in India, the national policy making body also opined that similar surveillance efforts should be conducted jointly by RNTCP and NACP in moderate and low HIV settings (states other than the ones mentioned above) of the country and findings presented before national policy decision. Given the heterogeneity of HIV epidemic in India across states/districts, it is not possible to define a cutoff value of HIV prevalence to decide if PITC among TB suspects is justified. Hence, it has been agreed that if the prevalence of HIV among TB suspects is as high as or greater than HIV positivity among the ICTC clients, then the strategy of PITC for TB suspects may be justified.

### Conclusions

Our study found that the prevalence of HIV among TB suspects was as high as that among TB patients and could be an important source of HIV case finding. The strategy of PITC in TB suspects was highly effective in detecting a large number of additional HIV cases and could be feasibly implemented with minimal additional workload on existing health staff. HIV testing of TB suspects aged 25–54 years demonstrated higher yield for a given effort. This study contributed towards making routine offer of HIV testing among TB suspects a policy in settings with high HIV prevalence. However, programme managers must scale up decentralized availability of HIV testing services and ensure availability of HIV testing kits in adequate numbers before launching this strategy.
